# AVOIDANT FILIAL PIETY IN KOREAN IMMIGRANT FAMILIES: WHEN DO WE TALK ABOUT MOM AND DAD?

**DOI:** 10.1093/geroni/igz038.990

**Published:** 2019-11-08

**Authors:** Joonsik Yoon

**Affiliations:** Syracuse University, Syracuse, New York, United States

## Abstract

Parental caregiving for older adults is a challenge to most families, not only because of intangible factors such as role-reversals in family dynamics, but due to the many practical difficulties involved. At the same time, family members are important sources of support and care even in wealthy countries with established and relatively efficient social services for the older population. For migrant families from East Asia, where adult children have emigrated while their parents have remained in the country of origin, the distance and the transnational nature of the family ties present additional complications to the challenge of providing parental care within a highly filial culture. In this qualitative study, first-generation Korean Americans were interviewed in open-ended, in-depth interviews about their experiences and concerns surrounding transnational parental caregiving. The results show that none of the study participants or their families plan for parental care. Although most respondents expressed concerns over potential future care needs of their parents, none of them had taken steps to address those concerns or discussed the matter with other family members, who were equally reticent to discuss it. This was even though many stated that they were specifically worried about how to care for their parents in times of need given that they lived far away in another country. The discussion explores why migrant families consistently avoid what they themselves acknowledge to be an important, even necessary, discussion their families ought to have.

